# The Relationship Between Performance and Trust in AI in E-Finance

**DOI:** 10.3389/frai.2022.891529

**Published:** 2022-06-21

**Authors:** Torsten Maier, Jessica Menold, Christopher McComb

**Affiliations:** ^1^Department of Industrial and Manufacturing Engineering, Kettering University, Flint, MI, United States; ^2^School of Engineering Design, Technology, and Professional Programs, The Pennsylvania State University, University Park, State College, PA, United States; ^3^Department of Mechanical Engineering, Carnegie Mellon University, Pittsburgh, PA, United States

**Keywords:** trust, robo-advisor, artificial intelligence, hidden Markov model, finance simulation

## Abstract

Artificial intelligence (AI) is fundamentally changing how people work in nearly every field, including online finance. However, our ability to interact with AI is moderated by factors such as performance, complexity, and trust. The work presented in this study analyzes the effect of performance on trust in a robo-advisor (AI which assists in managing investments) through an empirical investment simulation. Results show that for applications where humans and AI have comparable capabilities, the difference in performance (between the human and AI) is a moderate indicator of change in trust; however, human or AI performance individually were weak indicators. Additionally, results indicate that biases typically seen in human-human interactions may also occur in human-AI interactions when AI transparency is low.

## Introduction

Artificial intelligence (AI) is the development of computer systems to perform tasks typically requiring human intelligence (Copeland, 2020). AI is becoming fundamentally ingrained in our society as it powers unprecedented evaluation of large data, autonomous vehicles, purchase recommendations, and more (Helm et al., 2020). However, the effectiveness of such automated aids to complement human efforts is influenced by characteristics of the aid, like reliability and complexity and task factors like the type and goal (Parasuraman, 1997). Thus, understanding these factors is paramount to unlocking the full potential of AI.

Trust in human-machine interactions is complex and multi-faceted because of the multiplicity of influences affecting trust in automation. Chien et al. (2014) identified 42 items required for a standardized measure of trust in automation across three main constructs (performance expectancy, process transparency, and purpose influence) and three types of moderators (cultural technological contexts, individual, and cultural differences). Performance, in particular, has been identified as an integral element in understanding trust in human-automation interactions (Lee and See, 2004; Hoff and Bashir, 2015). Typically, performance has been measured as a percent reliability or through the overall quality of the interaction. While the study described in this paper does measure the performance of the robo-advisor through a standard quality performance metric (return-on-investment), it instead operationalizes an additional measure of performance: the difference between the return-on-investment of the user and the robo-advisor. In this metric, the robo-advisors performance is compared to the humans to understand what value it offered above and beyond the capabilities of the user. We expect that this additional comparison will provide further insight into the nature of the human-robo relationship, specifically the factors that contribute to or detract from the formation of trust.

Although performance and trust have been heavily studied in a variety of fields, no computationally derived predictive model defining the relationship between return-on-investment and trust in financial robo-advisors over time has been published. Such a model would allow designers and researchers to predict the impact of design decisions on the change in trust throughout a product's lifetime. In pursuit of this, the current work investigates the relationship between these two factors. Specifically, this work required human participants to complete a virtual investment simulation where they were asked to invest in stocks with the aid of an AI.

## Related Work

AI and machine learning have the potential to automate or aid humans in many facets of work. One such avenue is through the use of automated assistants (i.e., digital programs that use AI to aid user's in completing tasks). Automated assistants can be presented through a variety of modalities, such as virtual web-interfaces (i.e., chatbots) or physical devices (i.e., smart speakers). For example, currently, 20% of all households with Wi-Fi have a smart speaker (Bernard, 2018). Automated assistants are being used in applications ranging from e-commerce websites (Garcia-Serrano et al., 2004) to online finance (Bradesco | IBM, n.d.) to the design and analysis of Earth-orbiting satellites (Bang et al., 2018).

### Interpersonal Trust and Automation

Trust is defined as “the firm belief in the competence of an entity to act dependably, securely, and reliably within a specified context” (Grandison and Sloman, 2009). However, researchers must first understand which aspects of trust and what type of trust they are investigating to properly measure trust. For example, trustworthiness, trust propensity, and trust can be distinguished from one another (Colquitt et al., 2007):

- **Trustworthiness**: the ability, benevolence, and integrity of a trustee
- **Trust propensity**: a dispositional willingness to rely on others
- **Trust**: the attitude that an agent will help achieve an individual's goals in a situation characterized by uncertainty and vulnerability.

In the current work, participants accept vulnerability through investing monetarily in the AI which will, in turn, reinvest for them (a process which they could do themselves and which *may* result in no benefit to them). In user-AI relationships, both dispositional (trust in other persons or machines upon initially encountering them, even if no interaction has yet taken place) and history-based (founded on interactions between the person and another person or machine) elements of trust are potentially important (Merritt and Ilgen, 2008). In the current work, we focus predominantly on history-based trust as participants react to changes in the AI's performance and adjust accordingly. To understand how dispositional and history-based trust are formed, researchers must understand trust building processes. Lee and See define three processes that characterize how trust is developed: analytic, analogical, and affective processes (Lee and See, 2004).

- **Analytical** processes build trust through communicated knowledge, such as performance statistics
- **Analogical** processes build trust through experience
- **Affective** processes build trust through emotional connection.

Participants predominantly form analogical and analytical trust in the current work through repeated opportunities to evaluate the AI's automated performance and adjust. While the effects of affective trust are always present, even when working with automated aids, we expect the impact of this mode of trust to be minimal on the results as no new affective factors are introduced during the simulation. Because the results predominantly investigate the change in trust over time, factors that affect the initial trust state are mitigated in these results. For example, it is not anticipated that the emotional response to the color scheme of the simulation/AI interface will affect the change in trust over time.

Transparency is a key factor when investigating trust in automation (Jung et al., 2018a). After witnessing an automated error, participants of a study conducted by Dzindolet et al. (2003) were found to distrust automated aids. Moreover, participants distrusted even reliable aids unless an explanation for the error was provided. This speaks to the importance of transparency, the ability of the user to perceive the autonomous agent's abilities and develop an accurate mental model, which has been linked to mental workload and situational awareness (Chen et al., 2014). Mis-diagnosed errors, or errors or actions taken by an automated aid where the cause is incorrectly perceived by the operator, made by automation have been found to significantly impact user error and bias (Sauer et al., 2016). Additionally, previous work by Maier et al. (2020) found that a lack of transparency can lead users to incorrectly diagnose built-in functions as errors, leading to frustration. A lack of transparency has also been empirically linked to the slow adoption of robo-advisors (Zhang et al., 2021). However, increasing transparency is not the only way to improve trust in robo-advisors. Designers can also use user testimonials or establish a social presence through the use of instant chat or digital avatars (Zhang et al., 2021). Furthermore, the type of support provided by automation, such as robo-advisors, can be an important determinant for trust, with static agents providing continuous support and adaptive agents provide support specifically in critical situations. Adaptive automation has been shown to improve participants self-confidence, trust, and mental workload over static automation (De Visser and Parasuraman, 2011a).

Trust is an important concept within finance because “financing is nothing but an exchange of a sum of money today for a promise to return more money in the future” [pg. 1 (Sapienza and Zingales, 2011)] and this promise requires trust. Additionally, when working with finance agents (humans or robot) analogical trust built over repeated interactions is key (Sapienza and Zingales, 2011). For example, in the work presented in this paper, participants were shown the performance from their previous turn at the beginning of their next turn. This provides participants with repeated opportunities to assess and alter their trust.

### Measuring Trust

Within research, surveys such as the HRI (Human-Robot Interaction) Trust Scale are the most common method for measuring trust in automation (Yagoda and Gillan, 2012). However, longitudinal studies that use surveys have been found to suffer from panel conditioning (Lynn, 2009). This is “the possibility that survey responses given by a person who has already taken part in the survey previously may differ from the responses that the person would have given if they were taking part for the first time” [pg. 9 (Lynn, 2009)]. However, simulations allow researchers to reproduce authentic use-cases that elicit trust-related responses while controlling for a variety of otherwise uncontrolled variables and subsequently analyze the data to investigate the empirical implications of those variables on trust. For example, Calhoun et al. (2019) used a simulation environment to model the effect of pre-cursors of interpersonal trust on trust in an automated aid. In the study presented in this paper, we use a simulated financial scenario to measure or control the investment opportunities and AI behaviors experienced by the participants and measure their impact on participant's trust. Using monetary investment as a metric for measuring trust is a common method for studying and validating trust (Cochard et al., 2004; Buchan et al., 2008; Houser et al., 2010). For example, the Investment Game is a common 2-player scenario where monetary investment is used as a metric for trust (Evans and Revelle, 2008). In this scenario, a sender is given $10 and decides how much to invest, with the option to keep what they don't invest. The receiver is given triple the amount the sender invests. Finally, the receiver decides how much money to return to the sender or keep for themselves. The current work uses a similar method. Participants can send money to the AI which will invest it for them and return the earnings to them. However, the performance or expected rate of return is not known for the AI; thus, participants must discern whether they believe the AI will generate greater earnings than investing themselves through repeated interactions. This enables the researchers to evaluate, track, and investigate trust throughout the experiment.

### Automated Aids in E-Finance

The field of finance technology is leveraging AI to provide users with insights and to promote revenue (Park et al., 2016). For example, Bradesco Bank in Brazil uses a chatbot developed through IBM Watson to answer basic banking questions for their 65 million customers (Bradesco | IBM, n.d.). However, the most innovative and disruptive addition to financial technology have been robo-advisors: AI which actively assists in managing investments (Belanche et al., 2019). Robo-advisors are “digital platforms comprising interactive and intelligent user assistance components that use information technology to guide customers through an automated (investment) advisory process” and currently refers almost exclusively to financial investment (Jung et al., 2018a). However, these advisory systems could be applied to other fields, such as healthcare (Ferguson et al., 2010). The tasks undertaken by robo-advisors are usually one of two types: customer assessment, and customer portfolio management (Jung et al., 2018a). Customer assessment includes features such as questionnaires to measure risk attitude, preferences, goals, and special interests. Customer portfolio management includes features such as asset allocation, automated investment processes, and dynamic asset assessment. The robo-advisor presented to participants in the current work featured predominantly customer portfolio management tools.

These automated assistants can offer 24/7 assistance for a lower cost compared to traditional human advisors (Park et al., 2016). However, adoption of robo-advisors has been slow and is mediated by consumer attitudes such as perceived usefulness, familiarity with robots, and high expectations of transparency (Jung et al., 2018b; Belanche et al., 2019). Transparency refers to the degree to which a system's mechanisms are hidden from the user. Users are more satisfied and trusting of highly transparent systems (Jung et al., 2018b); this is typically achieved through cost transparency, process transparency, information transparency, or a combination of the three. The link between trust and transparency is particularly important between clients and their financial advisors because of the asymmetry in information (advisors are typically more knowledgeable) and interest (advisors can exploit information to take advantage of their clients) which can lead to distrust (Nussbaumer and Matter, 2011). Studies have also shown that trust plays a crucial role in technology acceptance and adoption (Bahmanziari et al., 2003; Srivastava et al., 2010; AlHogail, 2018). To counteract factors causing slow adoption rates (Jung et al., 2018a) developed design principles for robo-advisors that were categorized into the following four groupings:

- **Ease of interaction**: General requirements concerning the interaction with the artifact
- **Work efficiency**: Support the users' ability to achieve their goals in an adequate time-effort relation
- **Information processing and cognitive load**: Assist the user in information processing and understanding of the configuration
- **Advisory transparency**: Provide cost, process, and information transparency.

Notably, these design principles lack guidelines on trust. The research presented in this study aims to develop an computationally driven model of trust and performance which may aid in the creation of such design guidelines related to trust in robo-advisors.

### Research Question

AI is fundamentally changing the landscape of many fields by providing users with the ability to interact with AI through simple conversational language. However, our effectiveness at interacting with such automated aids is influenced by performance, complexity, task type/goal, and trust (Parasuraman, 1997). Trust, specifically, has been identified by the literature as a key component fundamental to understanding the human-AI relationship (Lee and See, 2004). Trust is also impacted by many factors. Performance (measured as return-on-investment in this study) has been identified as an integral element in understanding trust in human-automation interactions (Hoff and Bashir, 2015). However, no computational predictive model defining the relationship between return-on-investment and trust in financial robo-advisors over time has been published. Such a model would allow designers and researchers to predict the impact of design decisions on the change in trust throughout the use of a product's life. As such, the current work seeks to answer the following research question:

*What is the dynamic relationship between return-on-investment and trust between a human and financial robo-advisor?*

## Methods

In this study, participants completed a finance simulation that tasked them with investing money in simulated stocks. Participants were also given the option to give some (or all) of their money to an AI which would re-invest that money for them. The AI was designed to sometimes outperform (invest at a rate of return higher than the participant) and sometimes underperform (invest at a rate of return lower than the participant). Participants were asked to complete a pre-task survey covering financial literacy, risk aversion, and familiarity with the AI and upload their data to a cloud server after completing the simulation. In total, 45 participants completed this study. The following sections detail the methods and practices used.

### Experimental Design/Procedure

In this study, participants completed a pre-task survey followed by a virtual finance simulation that empirically measured their trust in an AI financial assistant. Participants were recruited through various email list-servs and through purposeful snowball sampling, a technique by which current participants are encouraged to recruit future eligible participants (Naderifar et al., 2017). Potential participants were directed to a webpage for screening and to receive instructions on how to participate. First, participants were required to complete a pre-task survey comprised of three sections:

1. Financial literacy: measuring how well an individual can understand and use personal finance-related information (Central Council for Financial Information., 2016; FINRA Investor Education Foundation, 2016). This section was comprised of five questions that covered interest rate, family budget management, life planning, selection of financial products, and the use of outside expertise and was based on large-scale financial literacy surveys released in 2016 (Central Council for Financial Information., 2016; FINRA Investor Education Foundation, 2016).
2. Risk aversion: the tendency of people to prefer outcomes with low uncertainty compared to outcomes with high uncertainty, even if the average outcome of the latter is equal or greater. Survey questions provided participants with a binary choice between participating in a lottery or guaranteed cash payments (Ding et al., 2010). The odds of payout and payout amount were different for each question.
3. Familiarity with AI: the degree to which a participant has prior knowledge and experience with AI. Familiarity with the technology has been shown to be a mediator of robo-advisor adoption (Belanche et al., 2019). Thus, two Likert scale questions were added addressing this factor.

After the pre-task survey, participants could begin the finance simulation. This simulation took place over 30 turns, each one representing 1 day. The number of turns was chosen to represent ~1 month of simulation time and ensure that participants could complete the experiment in a timely manner.

On Turn 1, participants began at the Overview Tab (see section Simulation Design for more information on the graphical interface). Participants started out with $5 to invest and on the Selection Tab they could choose to invest in four different stocks or give a portion of their money to the AI to invest for them. The simulation progressed to the next turn once 100% of their money had been invested and they chose to move on. During each turn, participants could review the previous turn's performance, examine newly generated information, and select which stocks to invest in for that turn. Nearly all information presented during the simulation was recorded for investigation. The primary information recorded from the simulation for this study includes:

1. The turn number to mark how far the participant had progressed through the simulation
2. The change in each stock from the previous turn (minimum −30%, maximum +30%)
3. The amount invested in each stock or the AI on each turn (minimum 0%, maximum 100%)
4. The rate of return of the human and the AI (based on a calculating how much was invested in each stock and by what percent that stock either increased or decreased).

After the final turn's selection was complete, participants were required to upload the supporting files generated by the simulation to a secure online portal maintained by the primary researcher. The purpose of this study was to measure the effect of performance on trust in a virtual finance scenario. Performance was measured as the difference in the rate of return between the human and the AI (measured each turn). Trust was measured as the total amount invested in the AI on a given turn.

### Simulation Design

**Figure 1:**
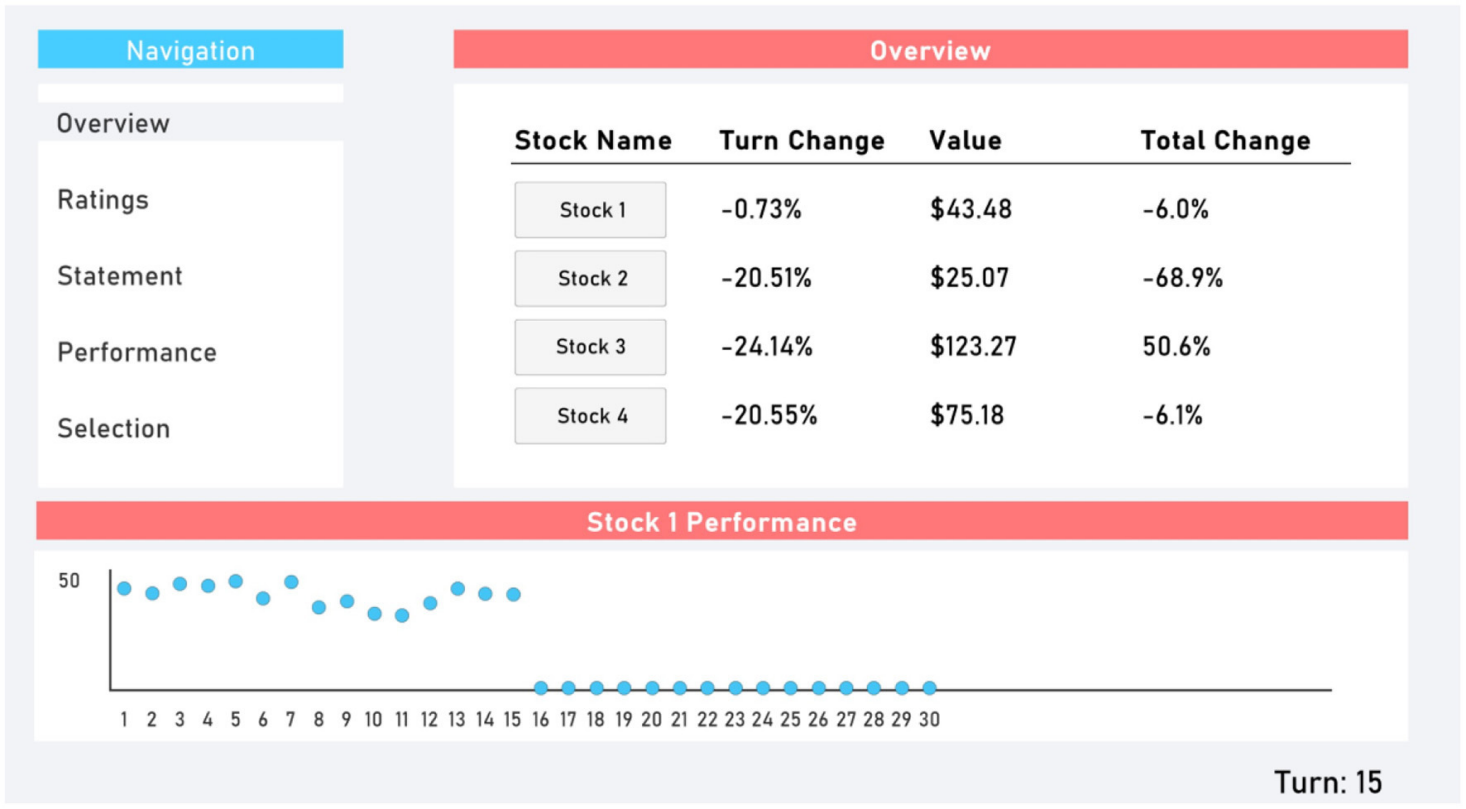
Overview tab.

The virtual finance simulation was designed using Unity2D. To participate, participants were required to navigate to a website where they could download the simulation executable file. At the conclusion of the simulation, participants were prompted to upload a batch of files that were generated in their download folder to an online portal that was automatically opened in their web browser. The simulation was comprised of a graphical user interface with five tabs: Overview, Ratings, Statement, Performance, and Selection. Participants could switch between these tabs freely. The Overview tab (Figure 1) provided information about the four randomly generated stocks. Participants were informed by what percent the stock had moved since the previous turn (randomly generated with a minimum of −30% and maximum of +30%), its current value, and by what percent it had moved since the beginning of the simulation. Participants could also see a chart of each stock's value up to that point in the simulation.

**Figure 2:**
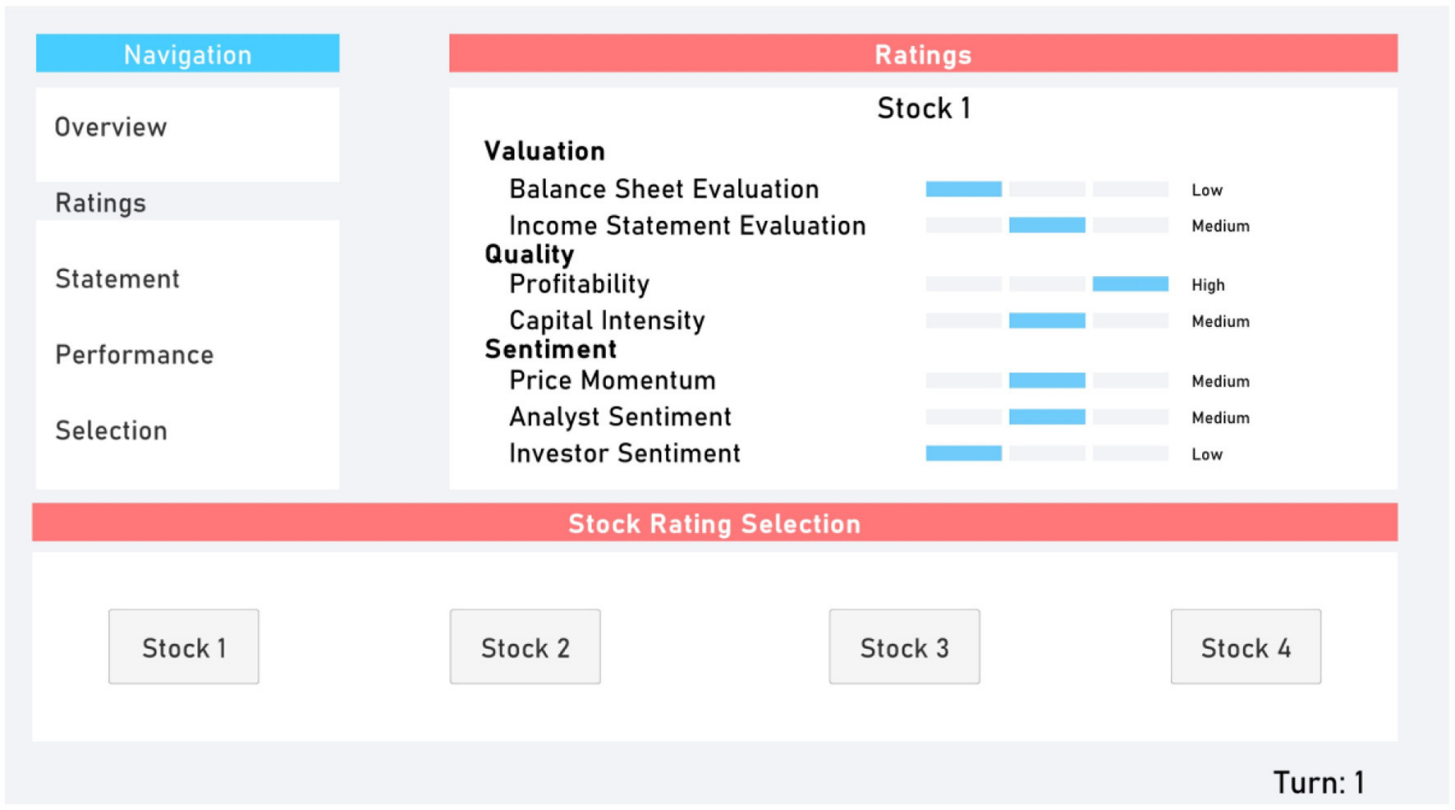
Ratings tab.

**Figure 3:**
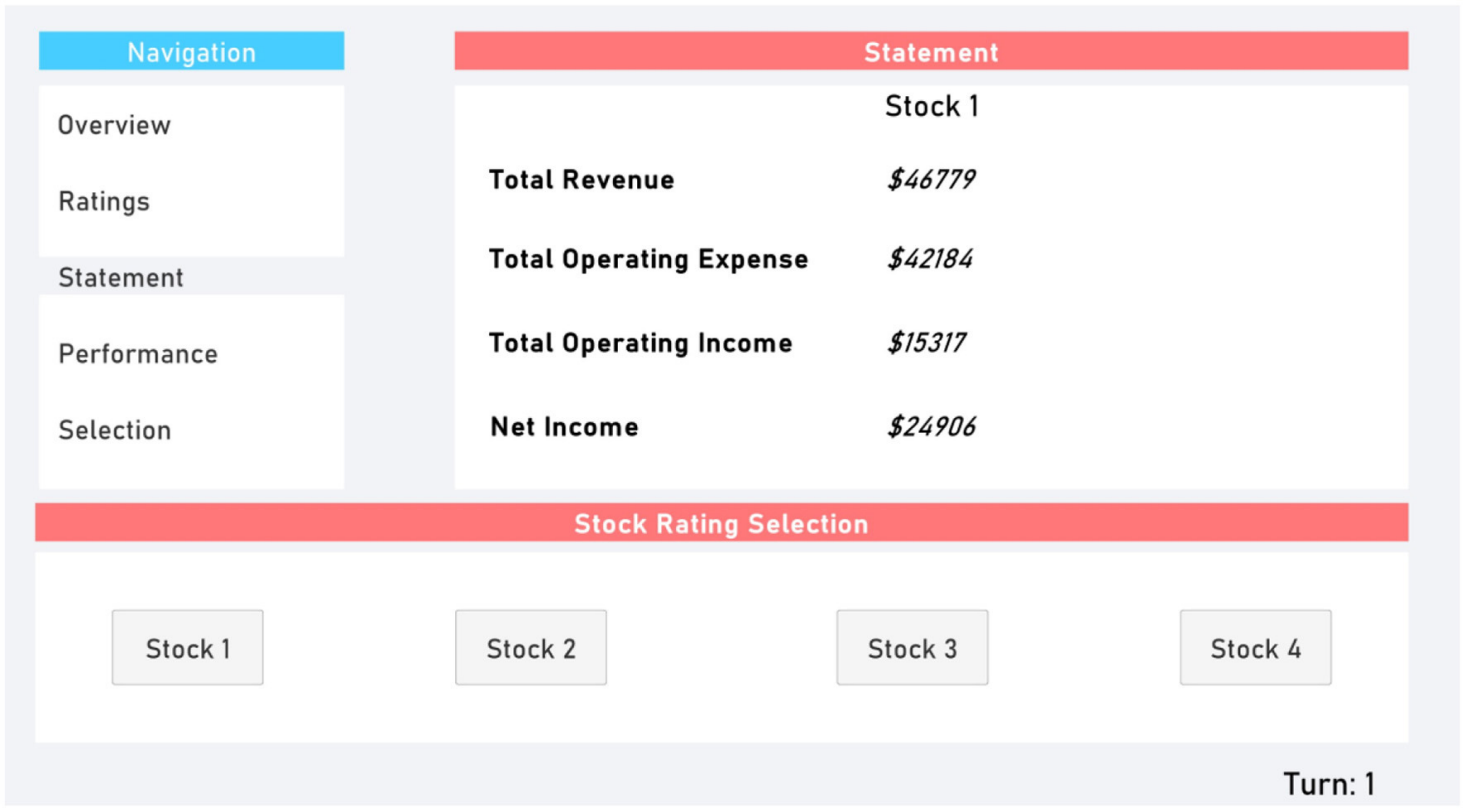
Statement tab.

The Ratings (Figure 2) and Statement (Figure 3) tabs provided participants with additional randomly generated data. This provided supplementary perceived depth and complexity.

**Figure 4:**
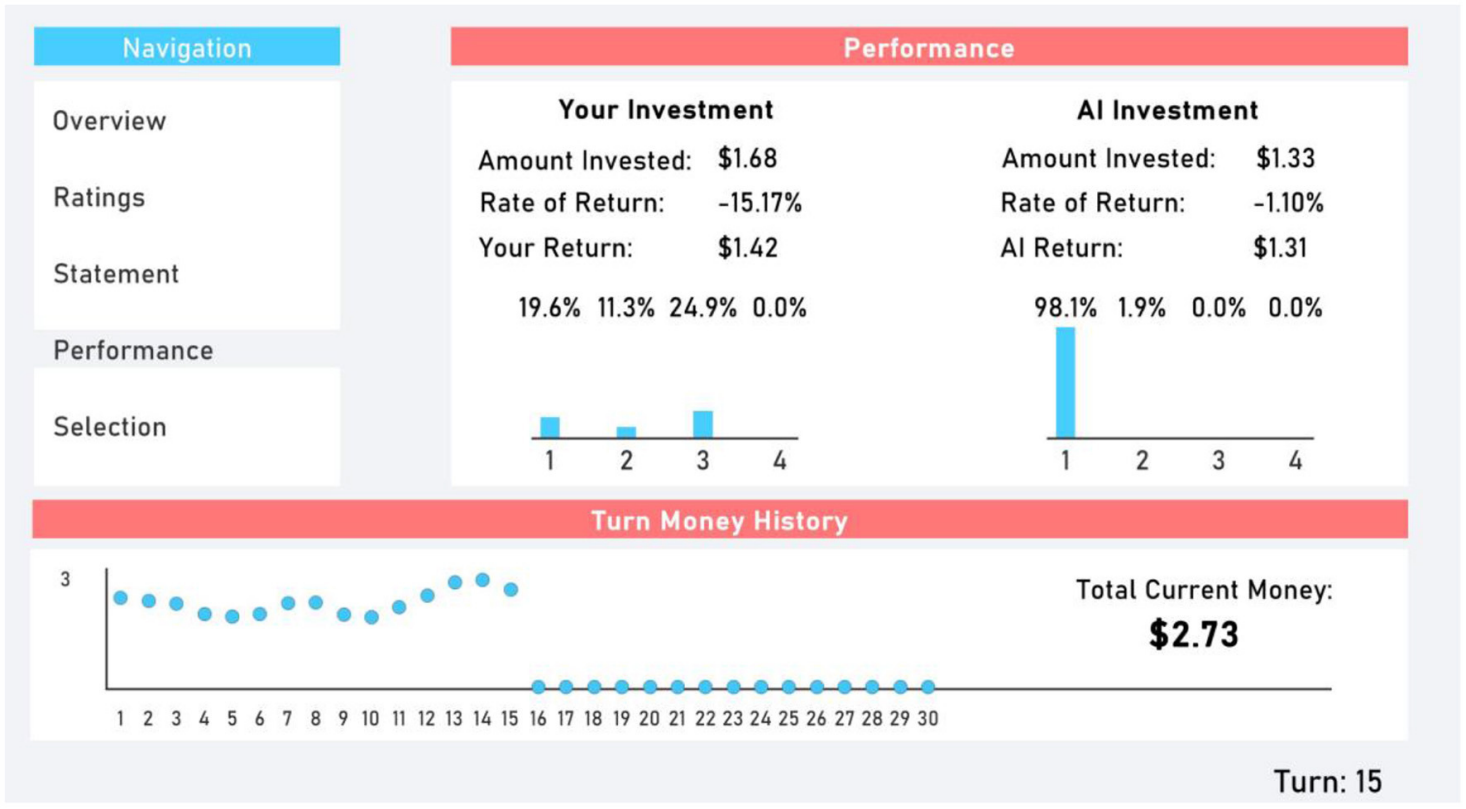
Performance tab.

Participants were sent to the Performance tab (Figure 4) after submitting their selection at the end of a turn. The Performance tab provided participants with information on their previous turn and the AI's previous turn's performance. This included the rate of return, total amount invested, and total amount returned. Additionally, the amount invested in each stock was presented through a bar chart.

**Figure 5:**
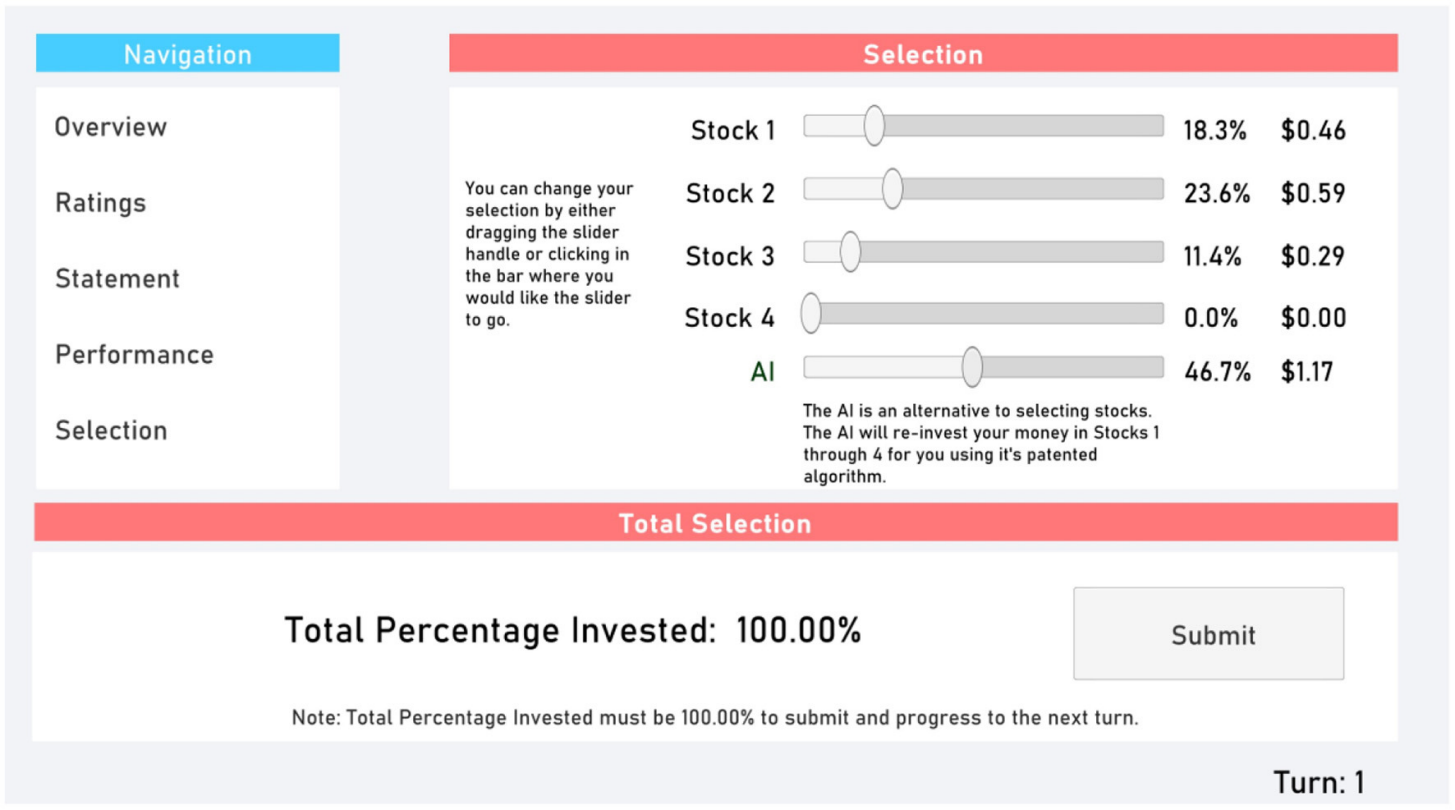
Selection tab.

Finally, the Selection tab (Figure 5) allowed participants to select which stocks to invest in. Additionally, they could invest in the AI. The total percent invested had to be 100% (i.e., participants could not save money between turns).

### AI Behavior

The intention of this study was to investigate responsive changes to trust and the effect of robo-advisor performance. Thus, the AI behavior was designed to be responsive to the participant's behavior. More specifically, the probability of failure of the AI was equal to the percentage investment in the AI on the Selection Tab. For example, if a participant invested 75% of their turn's money in the AI, there would be a 75% chance the AI would underperform the human. This AI behavior induced mistrust in participants who were highly confident in the AI and incentivized trusting the AI in participants who were highly skeptical of the AI. The methodology used in this study is based on prior literature that used controllable information to manipulate trust and study the effects on human-automation interactions (Körber et al., 2018) and was necessary to ensure data involving participants changing their trust in the robo-advisor (as opposed to keeping the same trust level for the duration of the simulation) occurred in each simulation. The AI underperformed by investing larger percentages in decreasing stocks and smaller percentages in increasing stocks than the human or overperformed by investing larger percentages in increasing stocks and smaller percentages in decreasing stocks.

### Participants

**Figure 6:**
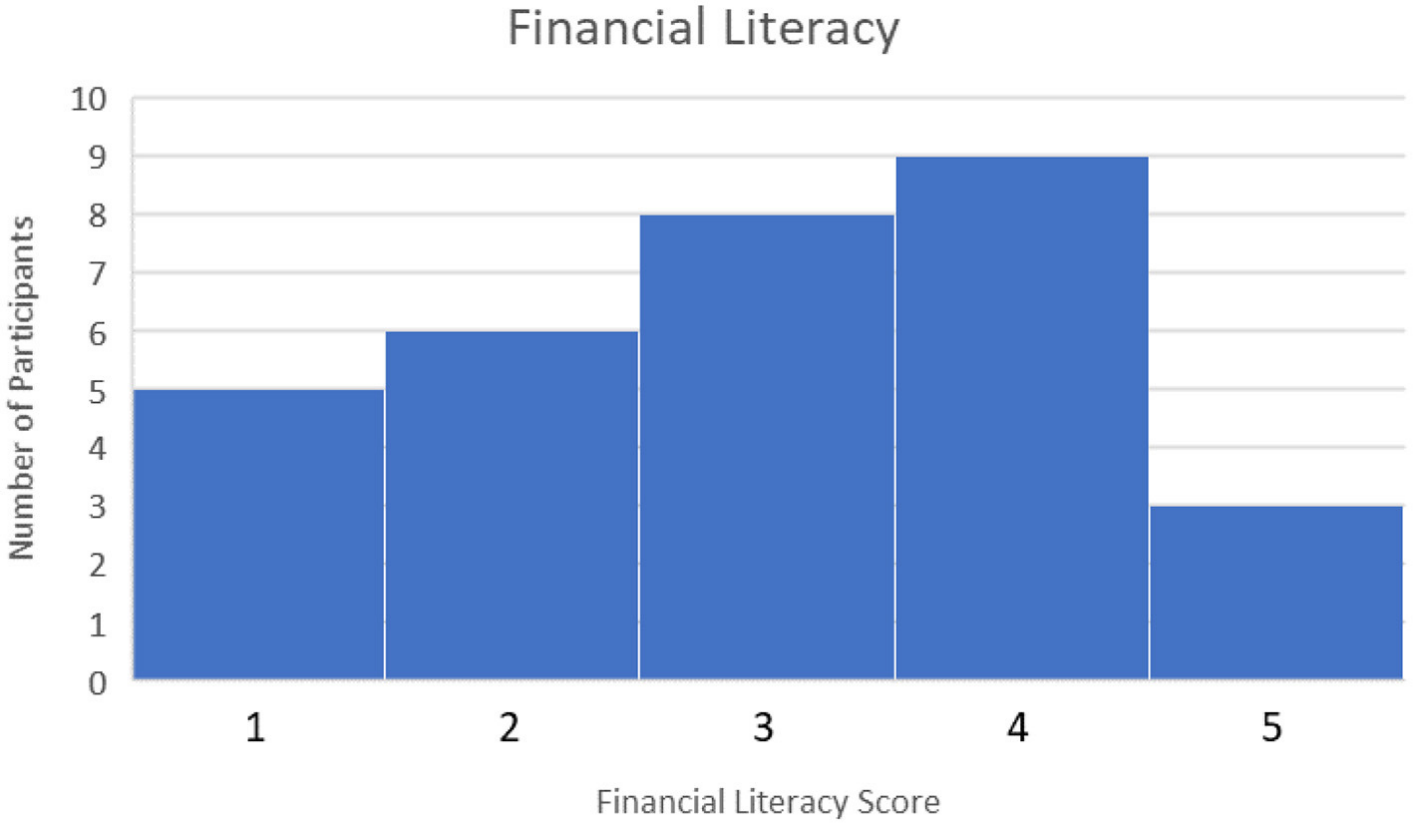
Financial literacy scores.

**Figure 7:**
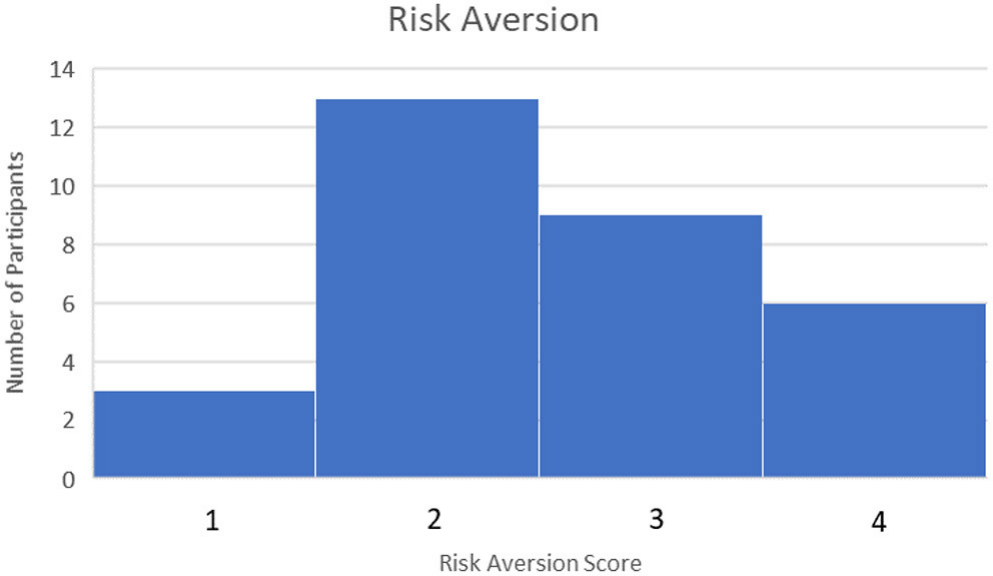
Risk aversion scores.

**Figure 8:**
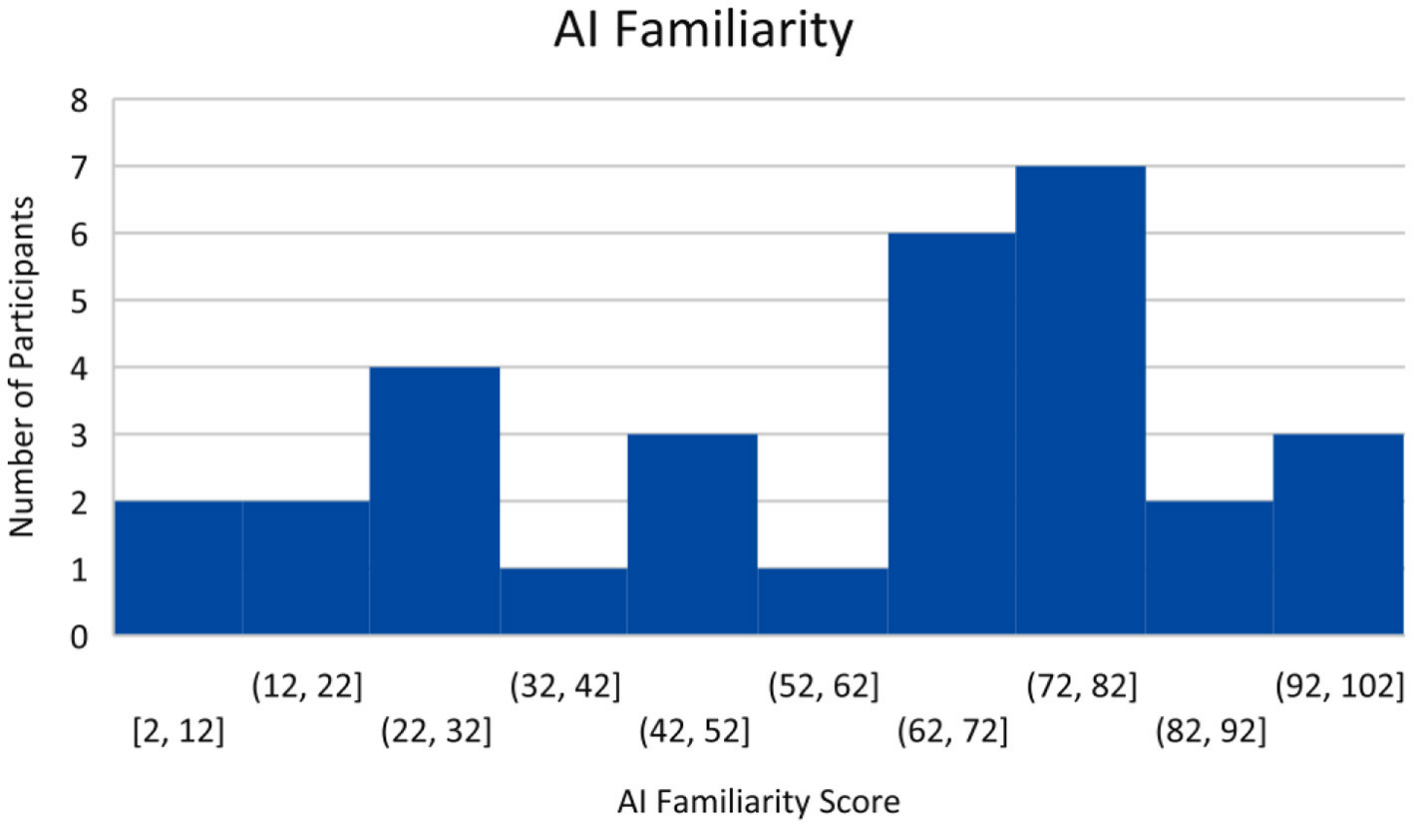
AI familiarity.

Forty five Participants Completed the pre-Task Survey and Finance Simulation and Uploaded Their Task Data to the Experiment Data Cloud Server. Participants Were Recruited Through Various Campus Email List-Servs and Through Purposeful Snowball Sampling, a Technique by Which Current Participants Are Encouraged to Recruit Future Eligible Participants (Naderifar et al., 2017). Participants were screened by age (18+ years or older) and language (fluent/literate in English only). Participants were paid a base rate of $5 for participating in the study. However, they could receive up to $5 additional dollars for ending the study with more money than they started with (the amount above the baseline determined how much additional money they received, up to a maximum of $10 total for participating). This ensured participants were “bought into” the outcome of the simulation and were motivated to perform their best. No demographic or identifiable characteristics were collected, other than an email to send compensation. However, the pre-task survey rated participants on their financial literacy, risk aversion, and familiarity with AI. Financial literacy was rated between 0 and 5 depending on how many questions were answered correctly with a higher score indicating higher financial literacy. The median score was 3. This can be seen in Figure 6. Risk Aversion was rated between 0 and 4 with lower scores indicating more risk averse individuals. The median score was 2. This can be seen in Figure 7. Finally, familiarity with AI was self-reported on a sliding scale from 0 to 100 with 100 indicating a greater familiarity with AI. The average was 59. This can be seen in Figure 8.

## Results

Multiple approaches were used when analyzing the data collected in this study. First, performance data was analyzed to investigate its impact on trust. Financial literacy, risk aversion, and AI familiarity were also analyzed for their impact on trust. However, no significant results were found, thus, these results are not discussed in the following sections. Second, data that changed over time was analyzed to identify behaviors that varied temporally. Finally, the data was analyzed to explore how participants transitioned between states.

### Trust vs. Performance

**Figure 9:**
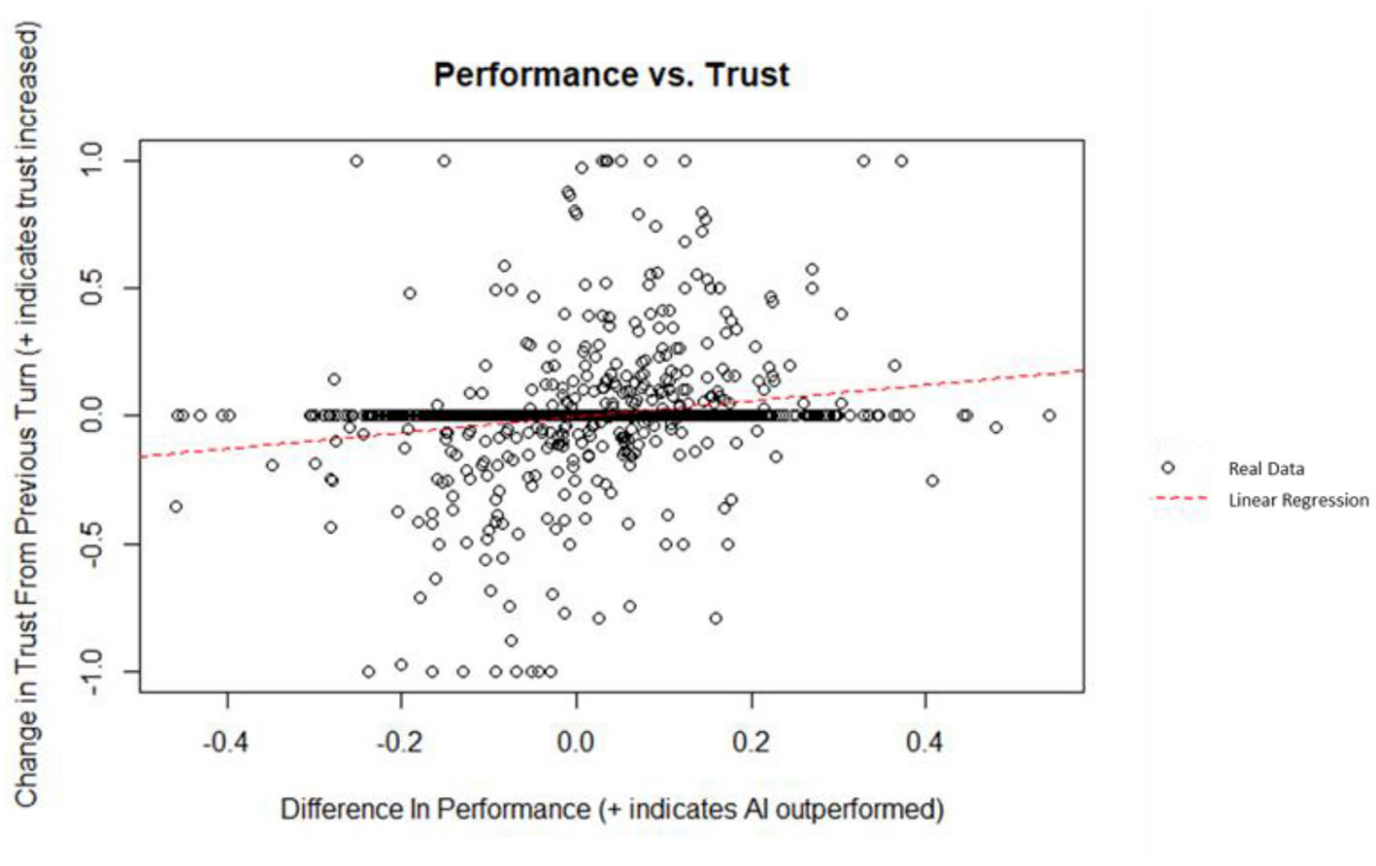
Difference in performance vs. change in trust.

A linear regression was used to predict the change in trust from the previous turn from the difference in performance between the AI and the human. Difference in performance did explain a significant amount of variance in the change in trust (*p* = 4.29 x 10^−12^; ${\text{R}}_{\text{adjusted}}^{2}$ = 0.03668). A plot of the data and linear best fit line can be seen on Figure 9. The regression coefficient indicated that an increase of one in the difference in performance corresponded, on average, to an increase in the change in trust by 0.311. The maximum change in trust is 1 (i.e., a participant changing from 0 to 100% AI investment); similarly, the minimum change in trust is −1. The theoretical maximum difference in performance is 0.6 (i.e., a participant who invested 100% of their money in a stock with a rate of return of 30% and the AI investing in a stock with a rate of return of −30%); similarly, the minimum theoretical difference in performance is −0.6. Although statistically significant, the low ${\text{R}}_{\text{adjusted}}^{2}$ of this regression means that performance is a weak indicator for change in trust in this simulation, according to Cohen's indices for coefficient of determination (Cohen, 1988).

This is, in part, due to the infrequency with which participants changed their trust (i.e., change the amount of money given to the AI to re-invest). On average, participants only changed their trust (i.e., allocated a different percent of their overall money for the AI to reinvest) every 3 turns.

**Figure 10:**
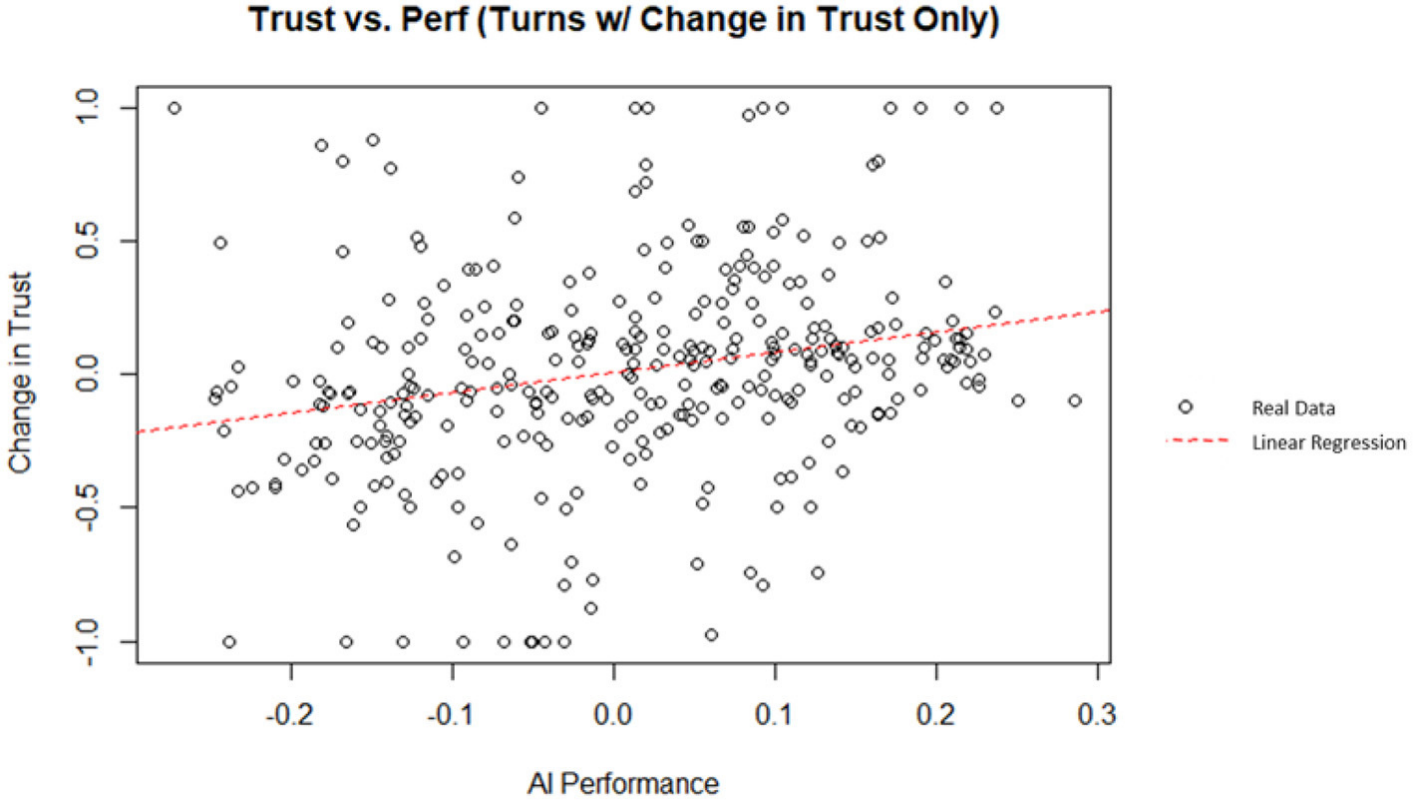
AI performance vs. change in trust (filtered for turns with a change in trust).

By removing the turns where no changes in trust occur, it is possible to isolate the decisions that resulted in a change in trust. These turns can then be analyzed to investigate if performance is a good predictor of the ensuing change in trust when participants chose to make a change. Additionally, the AI and human performance can be analyzed individually to investigate the predictive nature of each performance factor on their own. A linear regression was used to predict the change in trust from the previous turn from the AI performance (i.e., rate of return). AI performance did explain a significant amount of variance in the change in trust (*p* = 1.62 x 10^−05^; ${\text{R}}_{\text{adjusted}}^{2}$ = 0.05373). A plot of the data and linear best fit line can be seen on Figure 10. The theoretical maximum AI performance is 0.3 (i.e., the AI invested 100% of its money in a stock with a rate of return of 30%); similarly, the theoretical minimum AI performance is −0.3. The regression coefficient indicated that an increase of one in the AI performance corresponded, on average, to an increase in the change in trust by 0.759. However, according to Cohen (1988) indices for coefficient of determination, AI performance was a weak indicator for the change in trust in this simulation.

**Figure 11:**
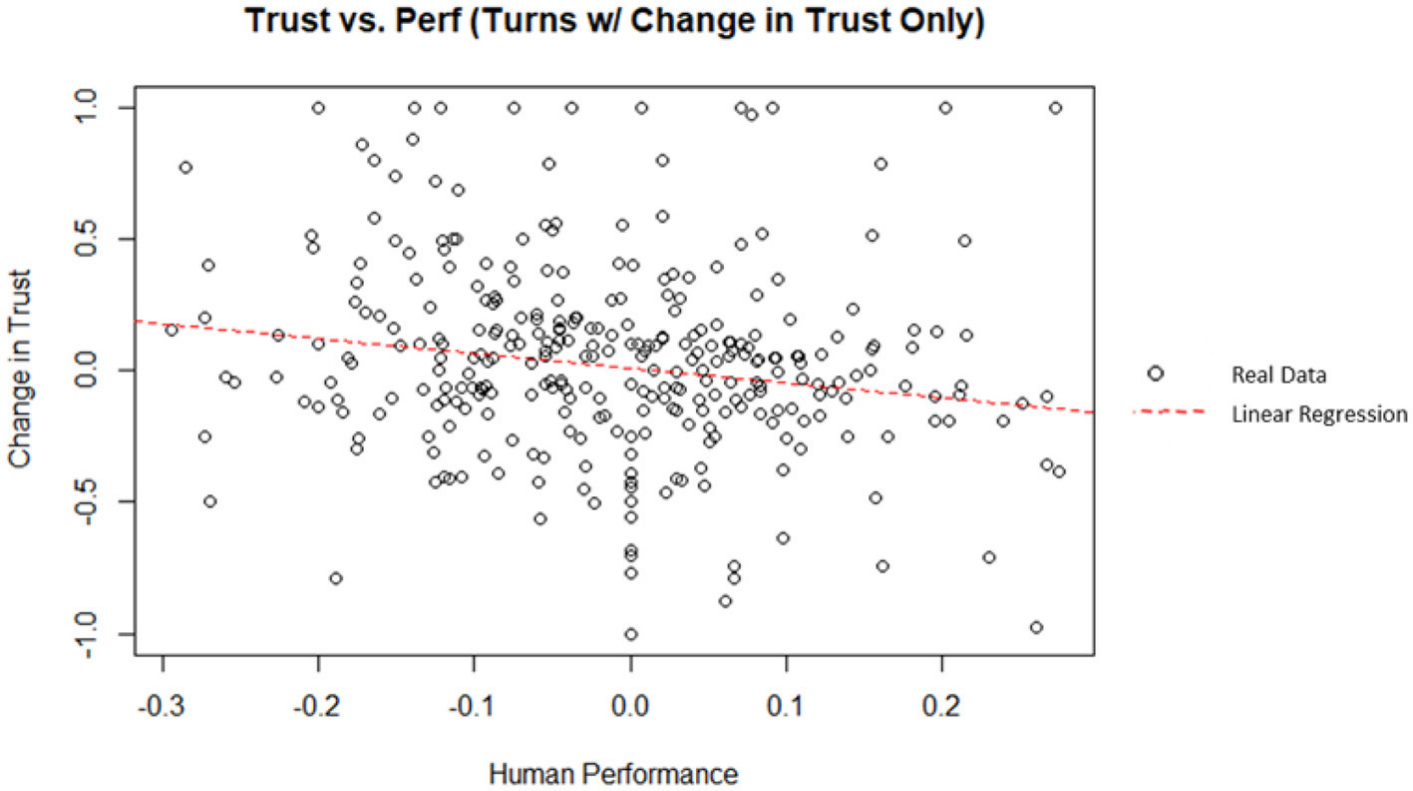
Human performance vs. change in trust (filtered for turns with a change in trust).

Similarly, human performance can be examined for its ability to predict change in trust. A linear regression was used to predict the change in trust from the previous turn from the human performance (i.e., rate of return). Human performance did explain a significant amount of variance in the change in trust (*p* = 0.00372; ${\text{R}}_{\text{adjusted}}^{2}$ = 0.02303). A plot of the data and linear best fit line can be seen on Figure 11. The theoretical maximum human performance is 0.3 (i.e., the human invested 100% of their money in a stock with a rate of return of 30%); similarly, the theoretical minimum human performance is −0.3. The regression coefficient indicated that an increase of one in the human performance corresponded, on average, to a decrease in the change in trust by 0.564. However, according to Cohen (1988) indices for coefficient of determination, human performance was a weak indicator for the change in trust in this simulation.

**Figure 12:**
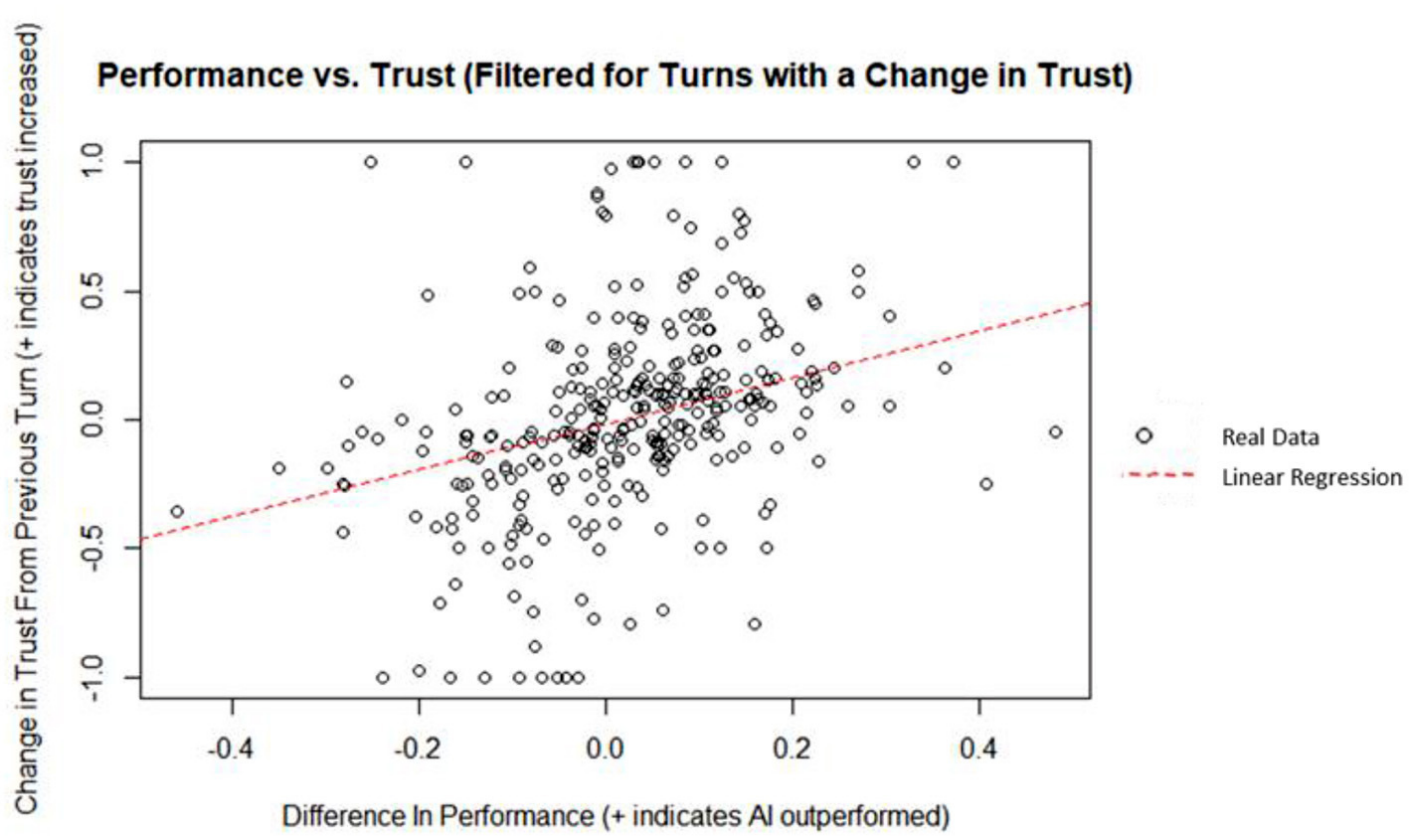
Performance vs. trust (filtered for turns with a change in trust).

Finally, human and AI performance can be used in conjunction by taking the difference of the two to improve the predictive power of the model. A linear regression was used to predict the change in trust from the previous turn from the difference in performance between the AI and the human. Difference in performance did explain a significant amount of variance in the change in trust (*p* = 5.37 x 10^−12^; ${\text{R}}_{\text{adjusted}}^{2}$ = 0.136). A plot of the data and linear best fit line can be seen on Figure 12. The regression coefficient indicated that an increase of one in the difference in performance corresponded, on average, to an increase in the change in trust by 1.15. By isolating the decisions that resulted in a change in trust and taking the difference in performance between the AI and the human, difference in performance was found to be a significant moderate predictor of change in trust based on Cohen (1988) indices for coefficient of determination.

### Changes to Behaviors per Turn

**Figure 13:**
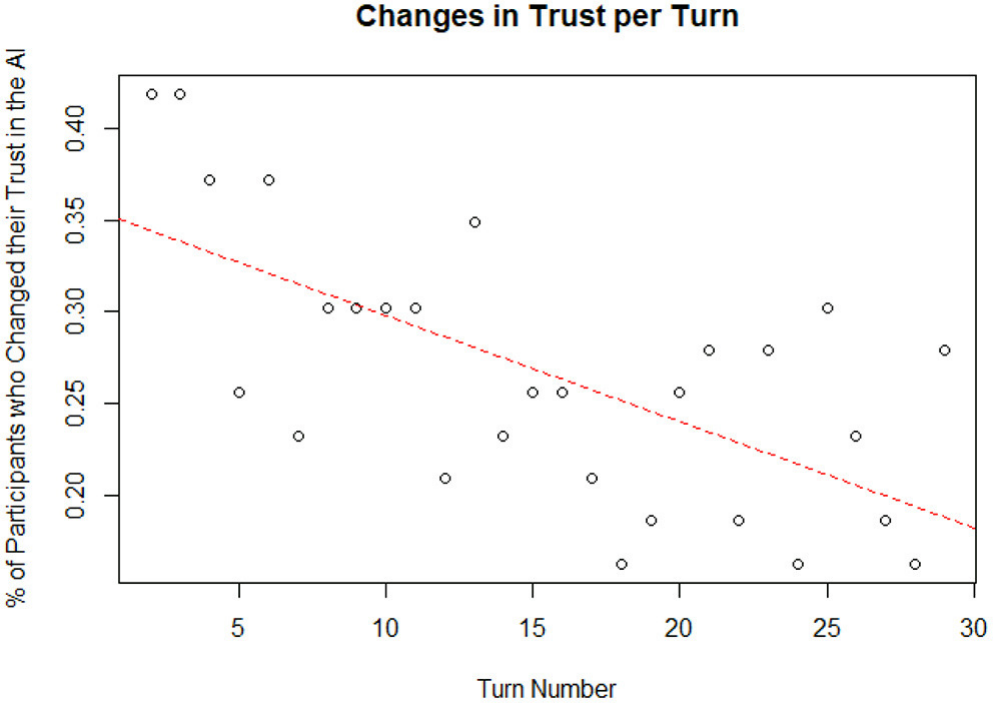
Changes to trust per turn.

The data was also analyzed by turn number to explore if there were any temporal trends. A linear regression was used to predict the change in the percent of total participants who altered their trust in the AI since the previous turn from turn number. Turn number did explain a significant amount of variance in the percent of participants who changed their trust in the AI (*p* = 1.77 x 10^−4^; ${\text{R}}_{\text{adjusted}}^{2}$ = 0.4013). A plot of the data and linear best fit line can be seen on Figure 13. The regression coefficient indicated that an increase of one in the turn number corresponded, on average, to a decrease in the percent of participants who changed their trust in the AI since the previous turn by 0.58%. Based on Cohen (1988) indices for coefficient of determination, turn number is a substantial predictor of the percent of participants who altered their trust in the AI since the previous turn. This may indicate that participants will continue to make fewer changes to their trust over time until eventually reaching a steady-state trust value; however, trends beyond 30 turns are hypothetical and should be investigated in future studies.

**Figure 14:**
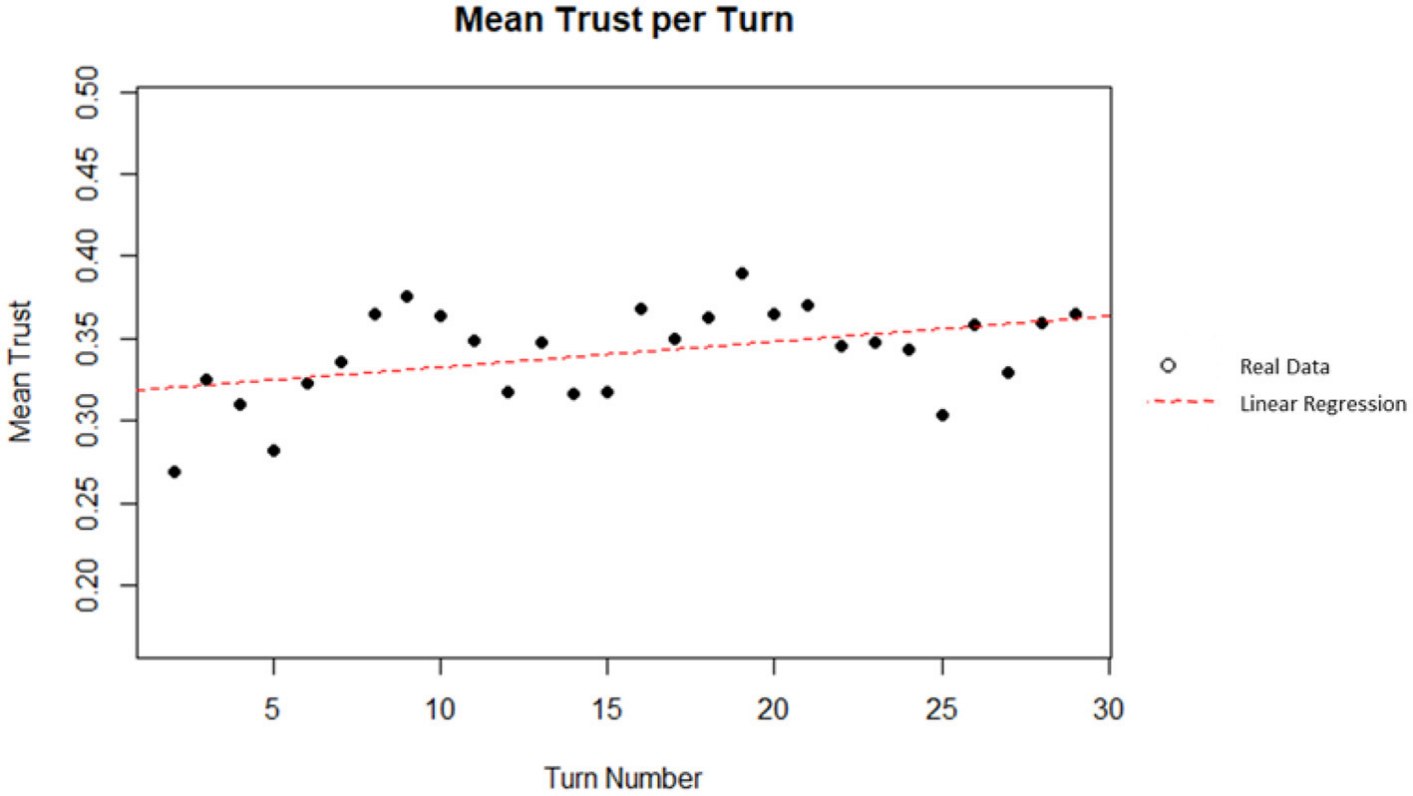
Mean trust per turn.

A linear regression was used to predict the mean trust (average percent of total money invested in the AI) from turn number. Turn number did explain a significant amount of variance in the mean trust (*p* = 0.02018; ${\text{R}}_{\text{adjusted}}^{2}$ = 0.1595). The regression coefficient indicated that an increase of one in the turn number corresponded, on average, to an increase in mean trust by 0.0015. This corresponds to an increase of 0.045 or 4.5% in mean trust after 30 turns. Based on Cohen (1988) indices for coefficient of determination, turn number is a moderate predictor of mean trust. A plot of the data and linear best fit line can be seen on Figure 14.

### Transition Matrices

To understand how the current status of trust and performance affect ensuing trust and performance values, the data is used to train a Markov chain (i.e., a process defined by states where the values of a selected state are dependent on the prior state). This entails defining each turn in the simulation as a state and linking them chronologically. Markov chains are used in a variety of fields such as statistics, computer science, and genetics (Asmussen, 2003). They have been shown to be effective tools at analyzing human behavioral data (McComb et al., 2017). Specifically, six states were defined for this work:

1. **Trust Steady/AI Outperformed**: Trust did not change from the previous turn and the AI had a higher performance than the human on that turn.
2. **Trust Steady/Human Outperformed**: Trust did not change from the previous turn and the human had a higher performance than the AI on that turn.
3. **Trust Increase/AI Outperformed**: Trust increased from the previous turn and the AI had a higher performance than the human on that turn.
4. **Trust Increase/Human Outperformed**: Trust increased from the previous turn and the human had a higher performance than the AI on that turn.
5. **Trust Decrease/AI Outperformed**: Trust decreased from the previous turn and the AI had a higher performance than the human on that turn.
6. **Trust Decrease/Human Outperformed**: Trust decreased from the previous turn and the human had a higher performance than the AI on that turn.

**Figure 15:**
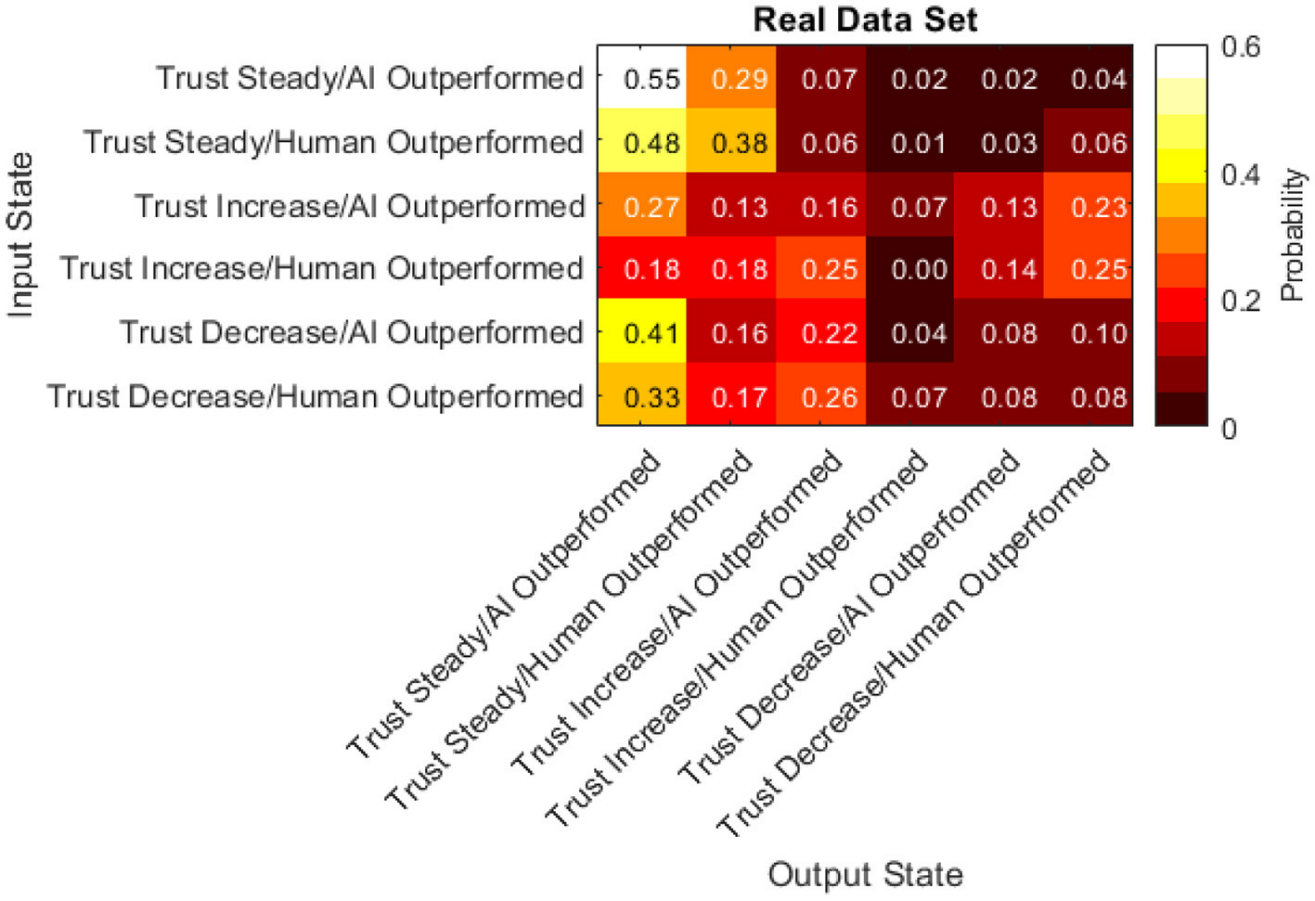
Transition matrix of real data set.

It is important to establish that the data satisfy the Markov property and are homogeneous across time (Bickenbach and Bode, 2001). Specifically, the Markov property was tested by training a first-order Markov chain and directly comparing it to several higher-order models using log-likelihood (McComb et al., 2017). In all cases, the tests did not reject the null hypothesis (*p* > 0.5 in all cases) indicating that the Markov property holds. Time homogeneity was verified by splitting the data temporally and comparing models trained on the different segments (Bickenbach and Bode, 2001). The null hypothesis was not rejected (Q^(T)^ = 45.84, df = 60, *p* = 0.08), so the time homogeneity assumption also holds. The transition matrix for the trained Markov chain model is shown in Figure 15.

**Figure 16:**
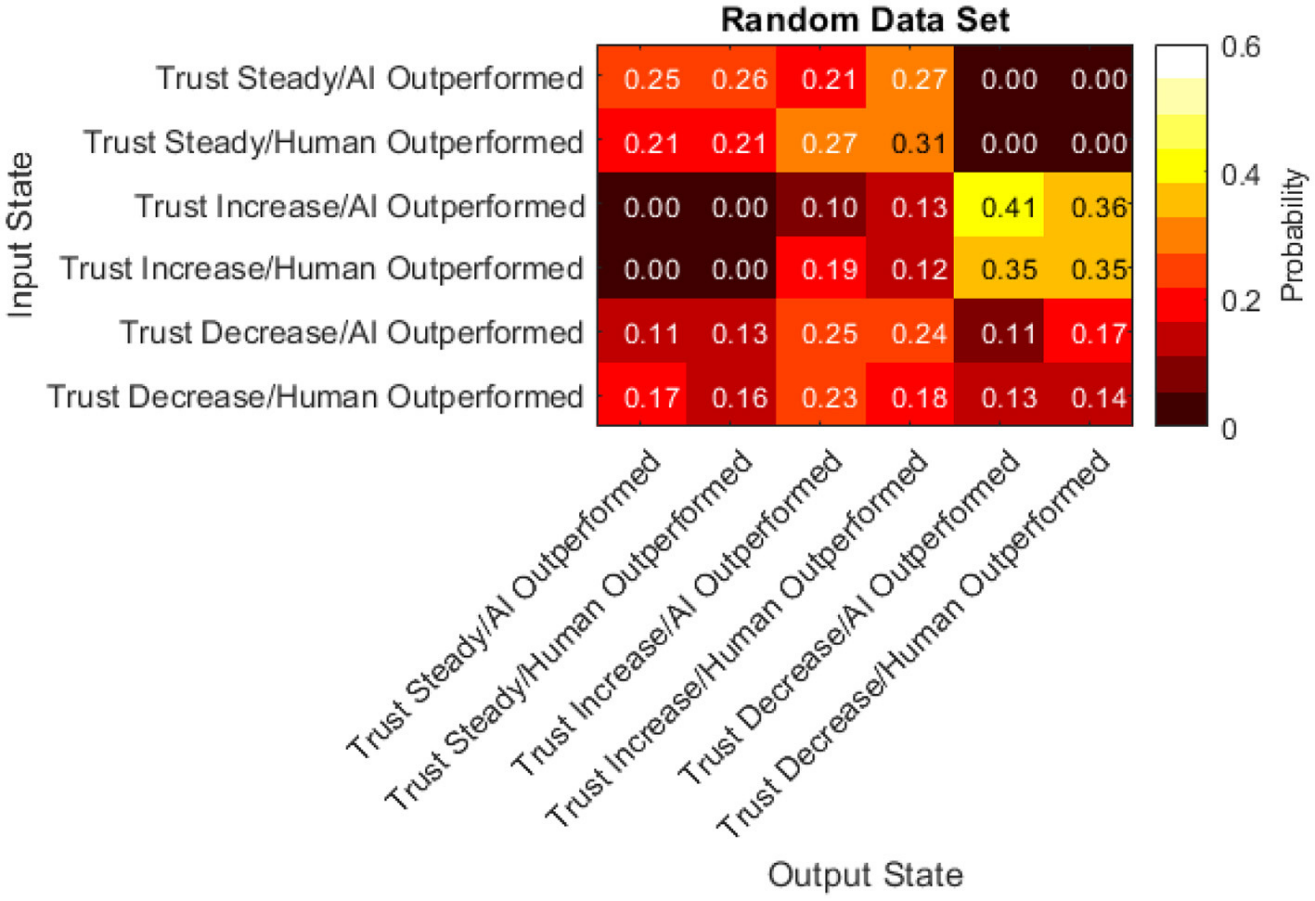
Transition matrix from randomly generated data set.

To fully understand which characteristics of the transition matrix could be attributed to human behavior and which characteristics could be attributed to the simulation or AI system, a data set equal in size to the real data set was randomly generated. This was accomplished by simulating a participant who, in each turn, invested in randomly-chosen stocks for random amounts regardless of any factors. This randomly generated data set was then used to produce another transition matrix seen in Figure 16.

Finally, the difference between the real data transition matrix and the randomly generated transition matrix was taken to pinpoint where the participants acted differently than random. A statistical comparison of these two matrices (Bickenbach and Bode, 2001) indicates a significant difference (Q^*^ = 3.52, df = 22, *p* < 0.001). Thus, examining the differences between them is warranted.

**Figure 17:**
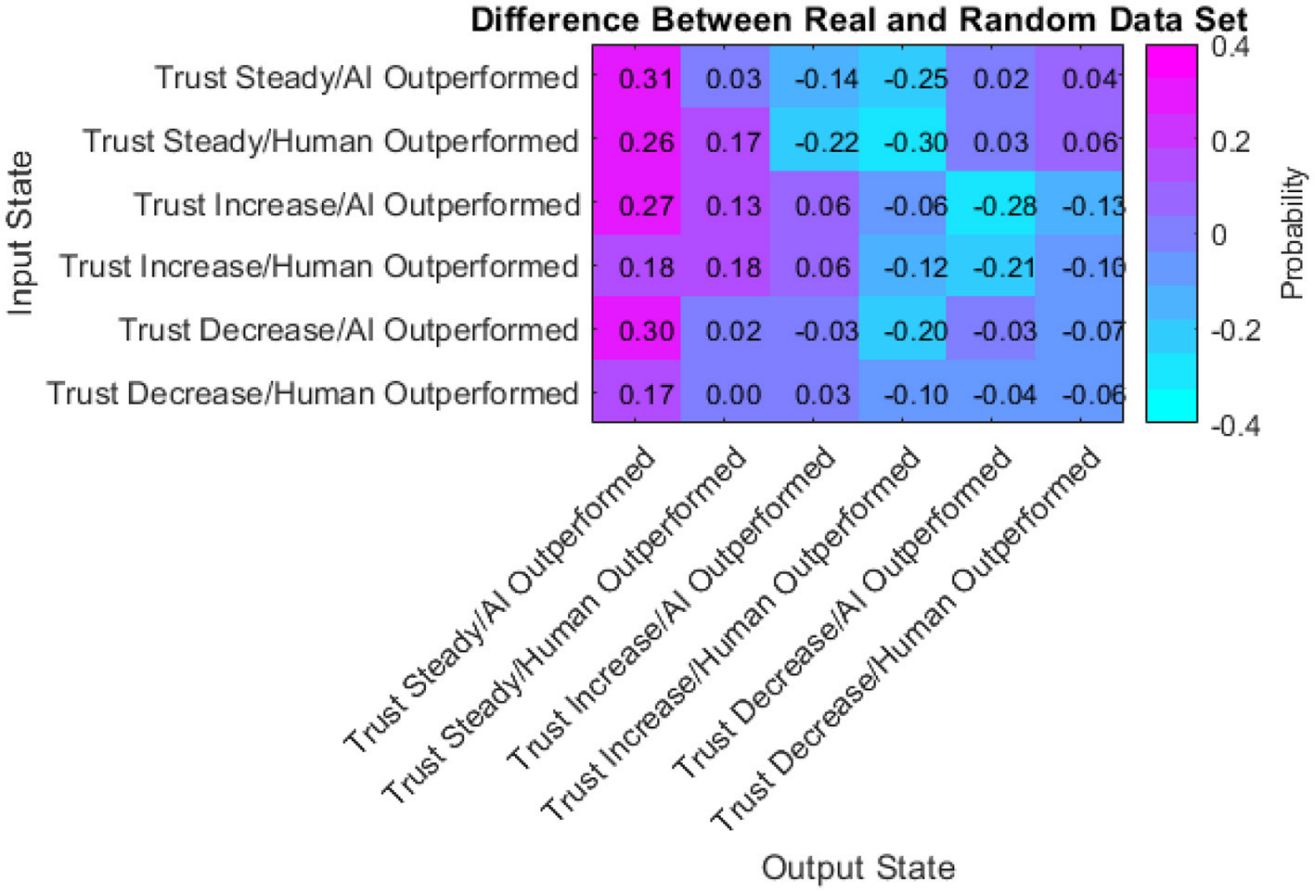
Difference between real and random matrix.

The probabilities within this matrix range from −0.30 to 0.31. Positive values indicate participants were more likely than random to transition from the input state to the output state. Negative values indicate participants were less likely than random to transition from the input state to the output state. The data presented in Figure 17 has a few notable characteristics:

- Participants were more likely than random to keep their trust the same after keeping it the same in the previous turn.
- Participants were less likely than random to increase their trust in the automated aid after a turn where they kept their trust the same.
- Participants were equally as likely as random to decrease their trust after a turn where they kept their trust the same
- Participants chose more frequently than random to not change their investment amount in the AI regardless of the previous turn.
- Participants chose less frequently than random to increase their investment amount in the AI when they outperformed the AI regardless of the previous turn.
- Participants were less likely than random to decrease their trust after a turn with an increase in trust.
- In half of the cells, participants acted with a likelihood similar to the random simulation (between −0.1 and 0.1).

This data described how participants acted in comparison to an impartial and random series of inputs to the simulation. The implications and conclusions drawn from these characteristics are described in section Transition Matrices.

## Discussion

### Impact of Performance on Trust

The analysis from the present work examines the link between performance and trust in the human-AI relationship. The findings indicate that the difference in performance between the human and the AI was a moderate predictor of change in trust on turns where participants chose to make a change in trust from the previous turn (i.e., invest a different amount of money in the AI than on the previous turn). Upon submitting a turn's selections on the Selection Tab, participants were routed to the Performance Tab where they could see their and the AI's rate of return from the previous turn. Findings suggest that participants compared their performance to that of the AI to inform the following turn.

The link between performance and trust found in this work supports previous literature in automation (Lee and See, 2004; Hoff and Bashir, 2015). The results from this study expand on previous literature in two key respects. First, participants did not make changes at each available opportunity (i.e., each turn) despite the AI being designed to outperform or underperform contrary to their current trust level (i.e., the probability of the AI performing worse than the human was equal to the percent of the total amount of money given to the AI on a turn). Instead, participants changed their trust, on average, every three turns. Participants may have needed multiple turns to theorize a pattern in the difference between their performance and the AI's. Importantly, this indicates that although performance can be used as an indicator to predict changes in trust on average, it may be not be well suited to predict when changes in trust *will or will not* occur. This is an important distinction because, as seen in the data collected in this work, people do not make a change at each opportunity they are presented with even when presented with evidence contradictory to their current strategy. Performance is not a strong indicator of those moments of inaction.

Second, unlike previous literature that uses only the AI's performance to make predictions (De Visser and Parasuraman, 2011b), the results of this study show that the difference between human and AI performance is a substantially stronger indicator of change in trust. For some applications of AI that perform incredibly complex tasks (e.g., large data computation) that humans are cognitively incapable of performing, this comparison between performance is moot and unlikely to be predictive of changes in trust. However, for applications such as finance management, this performance comparison is vital for predicting user's change in trust.

### Behavior Changes Over Time

The present work also examined the collected data for factors that changed over time (i.e., by turn number). By analyzing the percent of participants who made changes in trust per turn, it can be seen that the number of changes to trust tended to decrease over time. This may indicate that participants were trending toward a steady-state condition where they make very few changes, and their trust remains largely unchanged. However, behaviors past 30 turns are hypothetical and should be investigated in future studies. Additionally, it was shown that mean trust changed over time. This supports prior literature on analogical or history-based trust (Lee and See, 2004; Merritt and Ilgen, 2008). These theories state that trust is built through repeated experiences with automation. In this case, participants were found to increase their trust as the simulation progressed.

### Transition Matrices

Six states were defined to understand how the current status of trust and performance affect ensuing trust and performance values. These states were the combination of trust increasing, decreasing, or not changing and difference in performance being > or <0 (due to the random generation of stock changes, a difference in performance of 0 did not occur). By examining the probability of transitioning from one state to another through a transition matrix, we can draw valuable insights regarding the sequential dynamics of trust. Finally, by comparing these behaviors to randomly simulated data, we can differentiate which of these behaviors are due to human tendency.

First, participants were less likely than random to decrease their trust after a turn with an increase in trust. This may be indicative of an unwillingness or non-motivation to shift their course of action once committed. This type of behavior may be consistent with commitment bias (i.e., the tendency to remain committed to our past behaviors, particularly those exhibited publicly, even if they do not have desirable outcomes) (Hamza and Jarboui, 2012). Commitment bias has been shown to significantly impact monetary investment strategies, although not specifically linked to investment performance (Hamza and Jarboui, 2012).

Second, participants chose more frequently than random to not change their investment amount in the AI regardless of the previous turn. This indicates that participants did not alter their investment amount in the AI at each available opportunity even when presented with evidence indicating they should (i.e., being outperformed or outperforming the AI) and instead they decided to keep the amount they invested in the AI the same as the previous turn. This behavior is contrary to the expected optimal strategy. The findings from the transition matrix support cognitive behavior literature within finance that has found that investors are prone to biases and do not react to all forms of information with the expected strategy (Brosig, 2002; Cochard et al., 2004; Sharma and Jain, 2019).

Prior monetary investment simulations such as the investment game (Cochard et al., 2004) and the dictator game (Brosig, 2002) have also found human biases that prevent participants from executing optimal strategies. However, these simulations relied on two humans interacting and mistrust was often caused by not knowing the other participants intentions and motivations. In our study, the other agent is an automated aid. Thus, the researchers hypothesize that the effect of these biases are moderated by the level of transparency of the AI. In the present work, the AI's underlying functionality (i.e., how it decides which stocks to re-invest the participants money) is not revealed. This may mirror the ambiguous nature of not knowing the other human's motivation or rationale found in these human-to-human simulations. Additionally, transparency has already been found to be a key inhibitor to technological adoption (Jung et al., 2018b; Belanche et al., 2019) and trust (Chien et al., 2014). Theis hypothesis suggests that when AI transparency is low, biases and theories found in social psychology between humans may occur in human-AI interactions.

Finally, half of the transitions compared were found to be near-zero (between −0.1 and 0.1). Although participants were expected to respond to states where the AI outperformed them by increasing their trust in the AI and vice versa, this was often not the case. In the transitions found to be near-zero, participants did not follow this trend and instead responded no differently than random input. Many human biases can lead to non-rational decision making when investing [see Sharma and Jain (2019) for a review of these biases] and future studies should use qualitative methods such as think-aloud protocol or follow-up interviews to pinpoint the exact biases and effects resulting in this non-rational behavior.

## Conclusion

AI is having a profound effect on nearly every industry, including online finance. However, interacting with AI is affected by factors such as performance, complexity, and trust (Parasuraman, 1997). Thus, this study sought to examine the effect of performance on trust in a robo-advisor in a virtual empirical investment simulation. Forty five participants completed the simulation where they were tasked with investing money across 4 stocks and/or an AI that would re-invest in those same 4 stocks for them. The methodology of this simulation draws on prior literature that uses monetary investment simulations as a means of measuring trust and human behavior such as the Investment Game (Evans and Revelle, 2008) and the Dictator Game (Brosig, 2002). However, unlike these simulations, this study involved a human working with an AI and not a human working with another human.

The results of this study found that individually, neither AI nor human performance was a strong indicator for change in trust. However, the difference in performance between the AI and human was a moderate indicator for change in trust. This is an important distinction from prior literature that has found links between AI performance alone and trust in fields/applications where AI performance far exceeds human performance (Lee and See, 2004; De Visser and Parasuraman, 2011b; Hoff and Bashir, 2015). In applications, such as the investment simulation presented in this work, where AI performance is similar to human performance, people use both performance factors to inform their future decisions. Thus, to predict change in trust, both performance factors must be used in conjunction. Additionally, it was found that difference in performance is not a strong indicator for when trust won't change. This suggests that other metrics or factors should be used to predict periods of stagnant trust.

Furthermore, the results of this study support prior literature emphasizing the importance of analogical (Lee and See, 2004) and history-based trust (Merritt and Ilgen, 2008). Participants used their repeated experiences with AI to inform future decisions. Finally, the findings from this work indicate that when AI transparency is low, biases and theories found in social psychology between humans may occur in human-AI interactions.

Several avenues of future work may be informative. First, this work was conducted in a simplified simulation of an investment scenario. Although informative, future work must address scenarios which entail a greater degree of complexity. Second, we look at return on investment as the primary metric of interest. Again, while it was informative in this work, the breadth of alternative metrics used in investment advising (e.g., risk-adjusted return) may also be informative for understanding human-AI trust and interactions. Third, as additional metrics are addressed and additional scenario complexity is modeled, more detailed analytical techniques beyond simple linear regression may be necessary.

## Data Availability Statement

The raw data supporting the conclusions of this article will be made available by the authors, without undue reservation.

## Ethics Statement

The studies involving human participants were reviewed and approved by the Pennsylvania State University Institutional Review Board. Written informed consent for participation was not required for this study in accordance with the national legislation and the institutional requirements.

## Author Contributions

TM collected the data, performed analyses, and drafted the manuscript. All authors contributed to conceptualizing and designing the study, revising and editing the manuscript, and have read and approved the final version.

## Conflict of Interest

The authors declare that the research was conducted in the absence of any commercial or financial relationships that could be construed as a potential conflict of interest.

## Publisher's Note

All claims expressed in this article are solely those of the authors and do not necessarily represent those of their affiliated organizations, or those of the publisher, the editors and the reviewers. Any product that may be evaluated in this article, or claim that may be made by its manufacturer, is not guaranteed or endorsed by the publisher.
